# Targeting indoor residual spraying for malaria using epidemiological data: a case study of the Zambia experience

**DOI:** 10.1186/s12936-015-1073-9

**Published:** 2016-01-06

**Authors:** Jessie Pinchoff, David A. Larsen, Silvia Renn, Derek Pollard, Christen Fornadel, Mark Maire, Chadwick Sikaala, Chomba Sinyangwe, Benjamin Winters, Daniel J. Bridges, Anna M. Winters

**Affiliations:** John Hopkins Bloomberg School of Public Health, Baltimore, MD USA; Akros, Cresta Golfview Grounds, Great East Road, Unit 5, Lusaka, Zambia; Department of Public Health, Food Studies and Nutrition, Syracuse University, Syracuse, NY USA; US President’s Malaria Initiative, Washington, DC USA; Division of Parasitic Diseases and Malaria, Center for Global Health, Centers for Disease Control and Prevention, Atlanta, GA USA; National Malaria Control Centre, Ministry of Health, Government of the Republic of Zambia, Lusaka, Zambia; University of Montana School of Public and Community Health Sciences, Missoula, MT USA

**Keywords:** Indoor residual spraying, Targeted IRS, Focal IRS, GIS, Malaria

## Abstract

**Background:**

In Zambia and other sub-Saharan African countries affected by ongoing malaria transmission, indoor residual spraying (IRS) for malaria prevention has typically been implemented over large areas, e.g., district-wide, and targeted to peri-urban areas. However, there is a recent shift in some countries, including Zambia, towards the adoption of a more strategic and targeted IRS approach, in coordination with increased emphasis on universal coverage of long-lasting insecticidal nets (LLINs) and effective insecticide resistance management. A true targeted approach would deliver IRS to sub-district areas identified as high-risk, with the goal of maximizing the prevention of malaria cases and deaths.

**Results:**

Together with the Government of the Republic of Zambia, a new methodology was developed applying geographic information systems and satellite imagery to support a targeted IRS campaign during the 2014 spray season using health management information system data.

**Discussion/Conclusion:**

This case study focuses on the developed methodology while also highlighting the significant research gaps which must be filled to guide countries on the most effective strategy for IRS targeting in the context of universal LLIN coverage and evolving insecticide resistance.

## Background

The World Health Organization (WHO) launched the Roll Back Malaria Initiative in 1998, a global partnership with the goal to halve the burden of malaria. Since 2000, worldwide, the number of annual malaria infections has decreased by 26 % (173–128 million) with a concomitant 47 % reduction in mortality [1]. To continue this progress, proven interventions, such as rapid diagnostic testing (RDT), artemisinin-based combination therapy, intermittent preventive therapy for pregnant women, long-lasting insecticidal-treated nets (LLINs), and indoor residual spraying (IRS) are recommended [2, 3]. Universal coverage with LLINs is defined as one net per two people, and is recommended by the WHO for all people at risk of malaria [4]. To complement LLIN use, IRS has been scaled up in many African countries with the aim of supporting malaria control or elimination, depending on the underlying transmission. In 2014, a total of 90 countries, 42 in the African region, recommended IRS for vector control as a primary intervention for malaria [2].

IRS operates by either repelling mosquitoes from entering sprayed houses or by killing female mosquitoes that are resting inside houses after having taken a blood meal [5, 6]. IRS is most effective for endophilic and endophagic vectors, with maximum killing potency achieved when malaria vectors rest on IRS-treated inside walls [6, 7]. A ‘mass effect’ of IRS is thought to be obtained with high, e.g., >85 %, coverage of structures in a sprayed area [6]. Scientific evidence supporting this threshold is, however, limited and the combined impact in areas of high LLIN coverage is unclear from the few rigorous studies that have been conducted [8–10]. Furthermore, impact may be modified by transmission intensity and length of the malaria transmission season [11]. The WHO currently encourages full coverage of LLINs plus supplemental IRS, but more evidence is needed [12].

Historically, IRS has generally been implemented at district level or other similar, large-scale geopolitical unit. This approach is largely due to limited availability of data on the exact geographic distribution of households and IRS coverage at sub-district levels. This *status quo* approach presumably developed as most countries adopted a ‘blanket spraying’ strategy to target all eligible structures. The considerable challenges of delivering IRS, however, mean achievement of 100 % coverage is often unrealistic due to logistics, refusals, absent residents, and other factors, such that 85 % coverage is recommended by the WHO [6].

Increasing levels of insecticide resistance have forced IRS programmes to adopt insecticides costing more than triple the price of pyrethroids. For example, pyrethroid lambda-cyhalothrin costs ~$2–$3 per unit (sachet/bottle equivalent), whereas carbamate bendiocarb costs ~$12 per unit, while pirimiphos-methyl, a long-lasting organophosphate, costs ~$23 per unit. With one unit able to cover ~ three houses depending on size, the need to target resources to where they will have maximum impact becomes increasingly necessary in resource-constrained settings [13]. Considering that malaria transmission is highly heterogeneous within districts and is often focalized into hotspots (<1km^2^) [14–16], the strategy of blanket spraying in areas of universal LLIN coverage may be unnecessary and even cost-ineffective to achieve maximum gains in the reduction of malaria transmission, particularly in resource-constrained environments [2]. Unfortunately, limited policy and little data exist to inform the best strategies for targeted IRS to achieve maximum reduction in malaria transmission, particularly in areas of documented pyrethroid resistance and universal LLIN coverage. At this time, the WHO recommends only focal IRS in elimination settings to target remaining clusters or outbreaks of transmission [6]. However, sub-district targeting of non-pyrethroid IRS in low- to medium-transmission areas with universal LLIN coverage might be considered to mitigate pyrethroid resistance and drive down transmission in ‘hot spots’ [17].

Tools sufficient to manage targeted IRS campaigns must address three issues. First, the spatial location of all structures in a district must be mapped and the structures enumerated. Second, a robust targeting strategy must be developed to determine the size of the geographical units for which targeting is feasible or desirable to achieve the greatest impact with limited resources. Third, spray operators in the field must be guided to deliver IRS to targeted structures and record spray activities structure-by-structure to determine target area coverage. Other papers outline the use of freely available satellite imagery to determine the spatial location of all eligible structures [18], and forthcoming work will outline the development of a tool to guide spray operators in the field [19]. This paper focuses on the second aspect of targeted IRS campaigns: the need to develop robust targeting methodologies for IRS. Critical issues that remain to ensure effective and efficient IRS planning and implementation are outlined.

## Case description

### IRS operations in Zambia

In Zambia, IRS operations expanded from five districts in 2003, to 54 districts by 2014, supported in part by the US President’s Malaria Initiative (PMI). The 2011–2015 National Malaria Strategic Plan recommended IRS in high-risk areas (a minimum of 85 % of all targeted structures) with focalized IRS mounted in response to malaria surveillance data [20]. The methods to identify high-risk areas eligible for IRS were, however, not conclusively defined. Further, due to insecticide resistance and the need to change from pyrethroids to more costly organophosphates, PMI recommended the use of data to identify priority targets within previously sprayed IRS zones [3].

A targeted IRS strategy was planned for the 2014 spray campaign across 15 districts within Luapula and central provinces of Zambia, covering an area of 91,302 km^2^ and a population of 938,000 and 1,246,000, respectively. According to the 2012 Malaria Indicator Survey, malaria parasite prevalence by microscopy (or RDT) was 32.1 % (56 %) in Luapula and 8.5 % (12.8 %) in central province in children under five years of age [18]. To pilot the target strategy, ~275,000 structures in 154 health facility catchments within 15 districts were enumerated using satellite imagery and geographic information system (GIS) software. The methods used for enumeration of household structures have been described in detail elsewhere; in brief, these methods are 22 times quicker and ten times cheaper than standard ground-based enumeration [21]. A targeting methodology was then developed and is outlined below. Finally, IRS activity data were collected electronically with spatial coordinates through the use of a mobile to web data capture tool called mSpray^®^. Table 1 compares the methods used before and after this targeted approach was implemented.

**Table 1:** Comparison of planning processes for IRS pre- and post-targeted approach

	Prior to targeted approach	Targeted approach
IRS commodity planning	Commodities needed are estimated by multiplying the number of houses sprayed the previous year by an estimate of population growth	Commodities needed are calculated based on households mapped and targeted
IRS identification of structures	One month spent conducting a ground based census; stickers on doors identify households for spraying	One week spent enumerating structures from freely available satellite imagery through a desktop exercise
IRS targeting	Clusters of households identified for spraying based on geographic proximity to district health facilities and roads, and risk according to local knowledge	All households mapped, clusters of 25 or more households created, monthly health facility incidence calculated, clusters selected based on household density and clinical malaria incidence
IRS planning	Largely based on accessibility for spray teams and local knowledge/perception of malaria burden	Based on malaria incidence reported to health centers and population density, then subjected to local review
IRS implementation	Paper-based data recording	Mapped houses guide implementation. During IRS, all geographic positioning system (GPS) coordinates recorded in real-time allowing for ‘mop up’ of missed households
IRS surveillance	Malaria indicator survey (MIS) provides data on whether houses were sprayed. This survey occurs once every 3 years	All GPS coordinates for sprayed and unsprayed houses recorded for analysis

### Targeting

In previous spray seasons in Zambia, district healthcare workers and GRZ personnel met to discuss where spraying should occur. Spray planning based on this method has relied on local knowledge of malaria transmission, population locations and perceived risk. The process does not necessarily rely on routine health system malaria data, which despite its problems, in Africa it is often the only source of data available for evidence-based planning [22]. As a result, IRS has often been biased towards easily accessible high-density populations (e.g., near roads or clinics) to maximize the number of structures sprayed. Furthermore, target areas are generally imprecisely defined, leaving the interpretation of target areas up to the implementing team on the day of spraying.

**Fig. 1:**
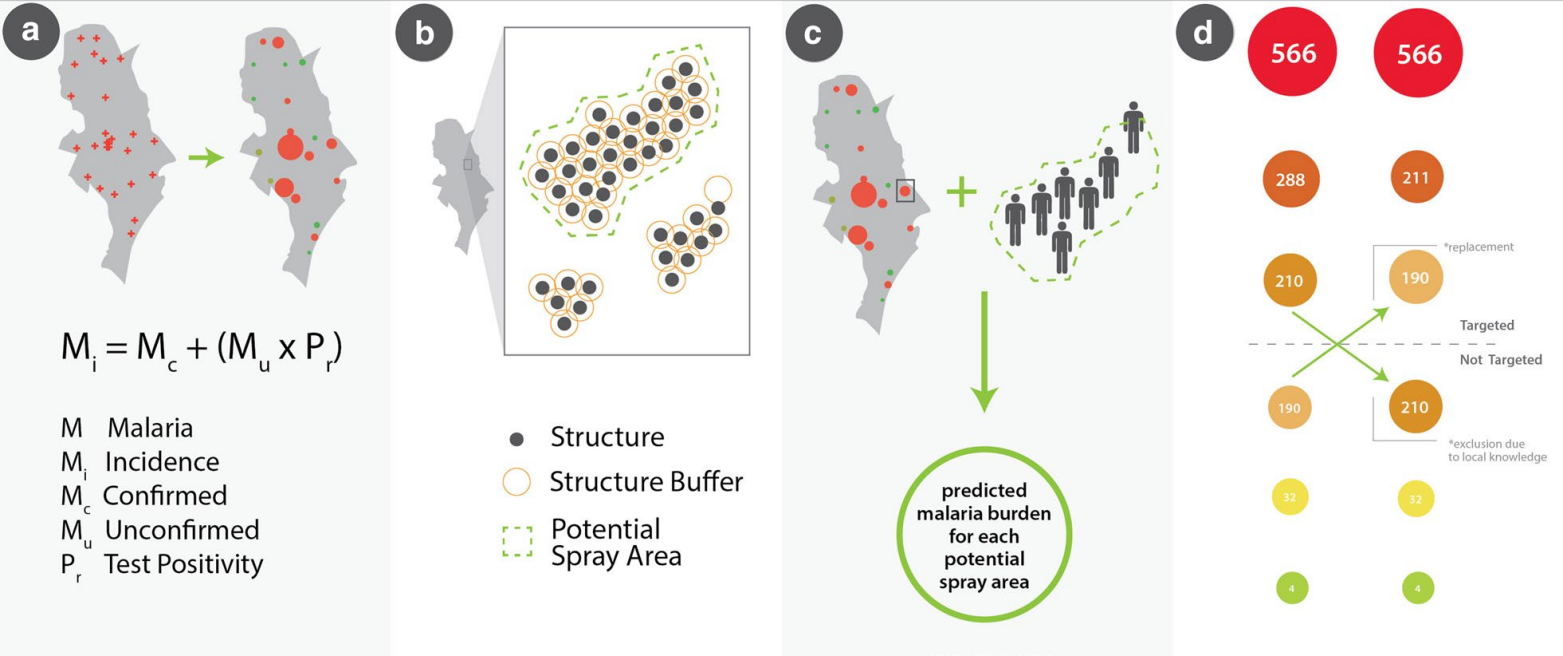
A targeting methodology to define target areas and assign ranking based on population and malaria incidence. **a** Health facility incidence is calculated from the confirmed and unconfirmed incidence data. **b** Target areas are defined as clusters of ≥25 structures that all lie within a contiguous area generated using 50-m buffers around each structure. **c** The malaria incidence per target area is generated using the estimated population and nearest facility incidence. **d** Target areas are ranked with final inclusion/exclusion based on local knowledge

For the 2014 spray season, the IRS budget was determined prior to target areas being defined and included sufficient funds to spray 125,731 structures across 15 districts. To prioritize target areas for IRS, and to stay within budget ceilings, a three-stage process was used to define all potential spray areas (PSAs) and to rank risk of malaria transmission for each (Fig. 1). First, a measure of estimated malaria incidence was calculated for each health facility catchment area by calculating the number of confirmed malaria cases each month plus the number of unconfirmed (clinical) malaria cases each month multiplied by that month’s test positivity (Fig. 1a). Second, a list of all PSAs was defined. This was achieved by identifying clusters of at least 25 enumerated structures; each enumerated structure in the cluster was located within 50 m of at least one other structure. A distance of 50 m and 25 structures was chosen to maintain operational efficiency: 50 m is the maximum distance walked by sprayers between structures within a settled area, and 25 structures is the minimum number of structures worth mobilizing a team to spray as per guidelines from the Zambian National Malaria Control Centre (NMCC). Third, an estimate of predicted malaria cases for each PSA was calculated by multiplying the number of estimated people in each PSA (assumed five people per household) by the estimated monthly malaria incidence at the nearest health facility. Finally, PSAs were ranked from highest to lowest, based on predicted number of malaria cases per month. District and provincial personnel reviewed the ranking and made minor modifications to the list based on local knowledge (e.g., site accessibility or seasonal movement of communities). Through this process, a final list of target areas for prioritization for IRS was generated.

## Discussion

Routine malaria incidence data were used to create an objective IRS targeting strategy via a method that is reproducible and relatively simple. In countries with a functional health management information system, this methodology does not necessarily require additional data collection. Based on the approach outlined, IRS can be targeted to high incidence, population-dense areas reducing distance necessary for spray teams to move from one structure to the next. Incorporating local knowledge and engaging district healthcare to confirm the selections and provide field-based guidance was also essential to this data-driven process.

Despite the ease of using health facility data to target IRS, there are limitations to the targeting approach used in 2014. For a number of reasons, routinely collected incidence data at the health facility may not always reflect the true underlying malaria risk. First, facility data is aggregated and therefore has very low spatial granularity. For example, a health centre catchment in Zambia may represent 8–10,000 people spread over 40 km^2^ or more. Malaria transmission is typically heterogeneous, but identifying sub-facility pockets with an elevated or depressed risk of transmission is not possible using health facility data at this time. Second, health facility data may be delayed or inaccurate due to recording errors or the lack of diagnostic confirmation [23–25]. The freely available malaria testing and treatment at government health centres likely encourages treatment, however, individual treatment seeking behavior may bias a health facility’s risk profile. For example, centres located at major transport nodes or those that have an above average reputation may find that they attract individuals from outside their catchment increasing the calculated incidence. Further, variation in the presence and variety of alternative sources of care (e.g., private clinics which do not report data to a central Health Management Information System (HMIS) may bias incidence measures.

Even in the presence of high quality health facility incidence data, there is a dearth of scientific evidence on how best to apply limited IRS to achieve maximum impact against malaria transmission. For the methodology described here, the number of expected malaria cases per target area was used to rank target areas from highest priority to lowest. This methodology biases the ranking toward larger target areas, which are financially and logistically easier to spray than an equivalent number of houses in multiple smaller target areas. However, it is unknown whether spraying these smaller areas with higher incidence rates would have a better impact on malaria transmission than spraying the larger areas with higher case counts.

A major benefit of this method was introducing a mapped and guided IRS approach to previously unmapped areas. However, the use of objectively defined target areas in some instances led to poorly understood target-area boundaries during field operations (Fig. 1). For example, what appeared to be a continuous stretch of adjacent households would sometimes be separated into two target areas, with one receiving IRS and the other not (owing to incidence and population factors in the ranking methodology). These anomalies should have been identified and rectified through scrutiny of the selected target areas during local review. In reality, it seemed this was not always achieved either through challenges with understanding the targeting methodology or translating the map. To rectify this, for the 2015 season, an evidence-based filter was developed to ensure that proximal target areas receive the same response when biologically appropriate and feasible.

## Conclusions

Few data exist on how to best move from the current implementation strategy (i.e., targeting IRS to maximize cost efficiency by focusing on the most accessible structures) to a strategy of targeting IRS based on epidemiological patterns. Such a data-driven approach is needed, particularly in areas of high LLIN coverage and/or insecticide resistance. The enumeration and operational aspects of targeted IRS have begun to be addressed through the use of satellite enumeration and geo-tagging IRS activities [21]. However, it is far from clear how best to identify the highest risk structures/areas to prioritize in a targeted approach.

Three ways are suggested to improve the accuracy of IRS targeting for future spray seasons. The first involves improving the quality and resolution of incidence data so that the most accurate, up-to-date, and least aggregated data are used to inform IRS targeting [26]. An example of this process may be seen throughout areas of Lusaka, central, southern and western provinces of Zambia, where malaria incidence is collected via mobile phone from a network of community health worker posts. With an average of eight health posts per health facility, community health workers have expanded care into the community and subsequently increased the spatial resolution of the HMIS data [27]. The second way to improve targeting of IRS is to incorporate malaria transmission maps that highlight entomological risk. Since the main goal of IRS is to kill and repel mosquitoes, spraying households near anopheline mosquito breeding sites, that are likely to have the highest mosquito density, may have a disproportionately higher impact on transmission. Targeting based on malaria incidence alone does not necessarily target households and populations that would benefit most from IRS. Predictive risk maps have been developed using satellite imagery and remotely sensed data to accurately characterize the location of mosquito breeding sites and high transmission risk areas [28], and further work should apply those findings to malaria intervention targeting. A third way of improving targeting is to include entomological data to account for insecticide resistance frequency and intensity, the primary vectors in the area and their seasonality and vector density. While entomological data are expensive to collect at fine scale, routine entomological surveillance systems are being employed in Zambia to build a better understanding of, and therefore better targeting of, the vector.

These three recommendations all focus on generating better data to provide a stronger platform to guide decision making for targeted IRS. However, more research is also needed to identify the specific targeting approach required to achieve the most effective IRS campaign. To that end, a comparison study of different targeted IRS approaches applied within various contexts is now being planned by this group and collaborators in order to generate evidence on the most effective and cost-efficient IRS strategies. In preparation for this study, baseline research is being collected in one of 15 districts that received targeted IRS in 2014 to understand the demographic and ecological factors associated with an effective, targeted IRS campaign.

In summary, advances in computing and GIS have opened the door to reassess and enhance the implementation of IRS. With limited malaria prevention and control tools available, it is essential that all available tools, including IRS, are used as effectively as possible. Further research to develop best-practice approaches for the implementation of IRS in environments of high LLIN coverage and also heterogeneous malaria transmission is necessary to inform malaria control programmes on the most effective and efficient IRS strategies to reduce malaria-related morbidity and mortality.

## References

[1] WHO (2014). World Malaria Report 2014.

[2] WHO Malaria Policy Advisory Committee and Secretariat (2014). Malaria Policy Advisory Committee to the WHO: conclusions and recommendations of fifth biannual meeting (march 2014). Malar J.

[3] President’s Malaria Initiative Strategy 2015–2020. PMI. 2014.

[4] WHO (2013). Recommendations for achieving universal coverage with long-lasting insecticidal nets in malaria control.

[5] Pluess B, Tanser FC, Lengeler C, Sharp BL (2010). Indoor residual spraying for preventing malaria. Cochrane Database Syst Rev.

[6] WHO (2013). An operational manual for indoor residual spraying (IRS) for malaria transmission control and elimination.

[7] Killeen GF (2014). Characterizing, controlling and eliminating residual malaria transmission. Malar J.

[8] Corbel V, Akogbeto M, Damien GB, Djenontin A, Chandre F, Rogier C (2012). Combination of malaria vector control interventions in pyrethroid resistance area in Benin: a cluster randomised controlled trial. Lancet Infect Dis.

[9] Pinder M, Jawara M, Jarju LBS, Salami K, Jeffries D, Adiamoh M (2014). Efficacy of indoor residual spraying with dichlorodiphenyltrichloroethane against malaria in Gambian communities with high usage of long-lasting insecticidal mosquito nets: a cluster-randomised controlled trial. Lancet.

[10] Protopopoff N, Wright A, West PA, Tigererwa R, Mosha FW, Kisinza W (2015). Combination of insecticide treated nets and indoor residual spraying in northern Tanzania provides additional reduction in vector population density and malaria transmission rates compared to insecticide treated nets alone: a randomised control trial. PLoS One.

[11] WHO (2014). Review of current evidence on combining indoor residual spraying and long-lasting insecticidal nets.

[12] RBM (2007). Report of the Fourth Meeting of the RBM Partnership’s Working Group on Scalable Malaria Vector Control (WIN).

[13] Chanda E, Mzilahowa T, Chipwanya J, Mulenga S, Ali D, Troell P (2015). Preventing malaria transmission by indoor residual spraying in Malawi: grappling with the challenge of uncertain sustainability. Malar J.

[14] Bejon P, Williams TN, Liljander A, Noor AM, Wambua J, Ogada E (2010). Stable and unstable malaria hotspots in longitudinal cohort studies in Kenya. PLoS Med.

[15] Bousema T, Griffin JT, Sauerwein RW, Smith DL, Churcher TS, Takken W (2012). Hitting hotspots: spatial targeting of malaria for control and elimination. PLoS Med.

[16] Carter R, Mendis KN, Roberts D (2000). Spatial targeting of interventions against malaria. Bull World Health Organ.

[17] WHO (2014). Guidance for countries on combining indoor residual spraying and long-lasting insecticidal nets.

[18] Ministry of health: Zambia National Malaria Indicator Survey 2012. 2012.

[19] The mSpray system. (http://www.akros.com/mspray).

[20] National Malaria Control Programme Strategic Plan 2011–2015. Government of the Republic of Zambia, Ministry of Health, 2011.

[21] Kamanga A, Renn S, Pollard D, Bridges DJ, Chirwa B, Pinchoff J (2015). Open-source satellite enumeration to map households: planning and targeting indoor residual spraying for malaria. Malar J.

[22] Rowe AK, Kachur SP, Yoon SS, Lynch M, Slutsker L, Steketee RW (2009). Caution is required when using health facility-based data to evaluate the health impact of malaria control efforts in Africa. Malar J.

[23] Chandler CIR, Jones C, Boniface G, Juma K, Reyburn H, Whitty CJM (2008). Guidelines and mindlines: why do clinical staff over-diagnose malaria in Tanzania? A qualitative study. Malar J.

[24] Githinji S, Kigen S, Memusi D, Nyandigisi A, Wamari A, Muturi A (2014). Using mobile phone text messaging for malaria surveillance in rural Kenya. Malar J.

[25] Hahn D, Wanjala P, Marx M (2012). Where is information quality lost at clinical level? A mixed-method study on information systems and data quality in three urban Kenyan ANC clinics. Glob Health Action.

[26] Chisha Z, Larsen DA, Burns M, Miller JM, Chirwa J, Mbwili C (2015). Enhanced surveillance and data feedback loop associated with improved malaria data in Lusaka, Zambia. Malar J.

[27] Larsen DA, Chisha Z, Winters B, Mwanza M, Kamuliwo M, Mbwili C (2015). Malaria surveillance in low-transmission areas of Zambia using reactive case detection. Malar J.

[28] Clennon JA, Kamanga A, Musapa M, Shiff C, Glass GE (2010). Identifying malaria vector breeding habitats with remote sensing data and terrain-based landscape indices in Zambia. Int J Health Geogr.

